# Human Monocyte-Derived Dendritic Cells Produce Millimolar Concentrations of ROS in Phagosomes Per Second

**DOI:** 10.3389/fimmu.2019.01216

**Published:** 2019-05-29

**Authors:** Laurent M. Paardekooper, Ilse Dingjan, Peter T. A. Linders, Alexander H. J. Staal, Simona M. Cristescu, Wilco C. E. P. Verberk, Geert van den Bogaart

**Affiliations:** ^1^Tumor Immunology Lab, Radboud University Medical Center, Radboud Institute for Molecular Life Sciences, Nijmegen, Netherlands; ^2^Princess Máxima Center for Pediatric Oncology, Utrecht, Netherlands; ^3^Department of Molecular and Laser Physics, Institute of Molecules and Materials, Radboud University, Nijmegen, Netherlands; ^4^Animal Ecology and Ecophysiology, Institute for Water and Wetland Research, Radboud University, Nijmegen, Netherlands; ^5^Department of Molecular Immunology, Groningen Biomolecular Sciences and Biotechnology Institute, University of Groningen, Groningen, Netherlands

**Keywords:** dendritic cells (DCs), reactive oxygen species (ROS), phagosomes, quantitative biology, NOX2

## Abstract

Neutrophils kill ingested pathogens by the so-called oxidative burst, where reactive oxygen species (ROS) are produced in the lumen of phagosomes at very high rates (mM/s), although these rates can only be maintained for a short period (minutes). In contrast, dendritic cells produce ROS at much lower rates, but they can sustain production for much longer after pathogen uptake (hours). It is becoming increasingly clear that this slow but prolonged ROS production is essential for antigen cross-presentation to activate cytolytic T cells, and for shaping the repertoire of antigen fragments for presentation to helper T cells. However, despite this importance of ROS production by dendritic cells for activation of the adaptive immune system, their actual ROS production rates have never been quantified. Here, we quantified ROS production in human monocyte-derived dendritic cells by measuring the oxygen consumption rate during phagocytosis. Although a large variation in oxygen consumption and phagocytic capacity was present among individuals and cells, we estimate a ROS production rate of on average ~0.5 mM/s per phagosome. Quantitative microscopy approaches showed that ROS is produced within minutes after pathogen encounter at the nascent phagocytic cup. H_2_DCFDA measurements revealed that ROS production is sustained for at least ~10 h after uptake. While ROS are produced by dendritic cells at an about 10-fold lower rate than by neutrophils, the net total ROS production is approximately similar. These are the first quantitative estimates of ROS production by a cell capable of antigen cross-presentation. Our findings provide a quantitative insight in how ROS affect dendritic cell function.

## Introduction

Reactive oxygen species (ROS) are a key component of the innate immune response, mostly in the neutrophil oxidative burst (1). When neutrophils, macrophages or dendritic cells phagocytose a pathogen, they rapidly activate the protein complex NOX2 (nicotinamide adenine dinucleotide phosphate oxidase 2) to generate superoxide anions in the phagosomal lumen (2). Activation is mediated by binding of the three cytosolic components of NOX2 (p40^phox^, p47^phox^, and p67^phox^) and the small-GTPase Rac1/2 with the two membrane components (p22^phox^ and gp91^phox^) (2). Superoxide anion can convert to other species of ROS, such as the highly reactive hypochlorous acid (HOCl) catalyzed by myeloperoxidase (MPO), which is responsible for the killing of ingested pathogens (3, 4). Because of the oxidative damage inflicted by bursts of ROS production, neutrophils usually die together with the ingested pathogen and are subsequently cleared by macrophages. It is estimated that superoxide generation in neutrophils can reach rates as high as 5 mM/s per phagosome for the duration of 10–15 min and then these rates rapidly decline within 30 min (5–8). Similarly, in macrophages the oxidative burst lasts for ~30 min after pathogen uptake (9).

In contrast, dendritic cells of the adaptive immune system exhibit prolonged ROS production, which plays a key role not only in the killing of ingested pathogens (10), but also in antigen presentation and cross-presentation (11, 12). Cross-presentation is the presentation of peptides derived from ingested antigens on major histocompatibility complex class I (MHC-I), which is normally restricted to endogenous peptides (13, 14). This process is essential for building cytolytic T cell immune responses to intracellular pathogens, certain viruses and various forms of cancer (15, 16). In order for antigens to be presented on MHC-I, they need to be preserved and allowed to leave the phagosome. Three mechanisms are described by which NOX2 promotes antigen cross-presentation. First, the dismutation of superoxide anion to hydrogen peroxide consumes protons that leads to alkalization of the endo/phagosomal lumen, which counteracts the V-ATPase, inhibits activation of lysosomal hydrolases with low pH-optima and thus preserves antigen for cross-presentation (11, 12, 17–19). Second, we previously showed that the ROS produced by NOX2 can oxidize lipids of the endo/phagosomal membrane leading to leakage of antigen from the lumen into the cytosol where it can enter the MHC-I pathway (20, 21). Finally, cysteine residues located in the catalytic core of certain cathepsin proteases can be oxidatively modified, which also prevents excessive degradation of antigen (22, 23). The last mechanism also alters the epitopes generated for presentation on MHC-II to helper T cells (24). The intracellular pathogen *Leishmania* evades host immunity by inhibiting antigen cross-presentation through disruption of NOX2 delivery to pathogen containing phagosomes (19). Since the process of antigen cross-presentation in dendritic cells is a much subtler process than that of pathogen elimination in neutrophils, lower ROS production rates can be expected, yet quantifications of ROS production rates in the phagosomes of dendritic cells are lacking.

Most ROS measurements rely on probes that change fluorescence upon oxidation, such as Amplex Red, H_2_DCFDA, or ADPA. Unfortunately, most, if not all, of these compounds are only responsive to certain ROS (25). However, most ROS can rapidly convert to various species, such as dismutation of superoxide anion to hydrogen peroxide and hydroxyl anion by Fenton chemistry. Moreover, as ROS are unstable, they are short-lived and readily react with proteins, lipids and nucleic acids (26), making quantitative detection by ROS-sensitive probes impossible. However, since the production of superoxide anion by NOX2 consumes oxygen in a 1:1 ratio, an increase in oxygen consumption rate (OCR) during the oxidative burst directly reflects the rate at which superoxide anion is produced (8). In this paper, we measured OCRs in the culture medium of human monocyte-derived dendritic cells in real time. These cells are derived from blood-circulating monocytes, and capable of both MHC-II presentation and MHC-I cross-presentation. Monocyte-derived dendritic cells are positive for dendritic cell markers CD11b, CD11c, CD14, HLA-DR, CD83, and CD86, and negative for monocyte and macrophage markers CD16 and CD68 (27), and, although their physiological role is unclear, they likely represent an inflammatory type of dendritic cell. Monocyte-derived dendritic cells were pulsed with opsonized zymosan particles, which are relatively monodisperse ~4 μm-sized yeast cell wall fractions that are readily ingested by phagocytosis, triggering NOX2 activity (20, 21, 28). Combined with quantitative microscopy, this allowed us to estimate the rate and duration of ROS production inside the zymosan-containing phagosomes of these dendritic cells.

## Results

**Figure 1:**
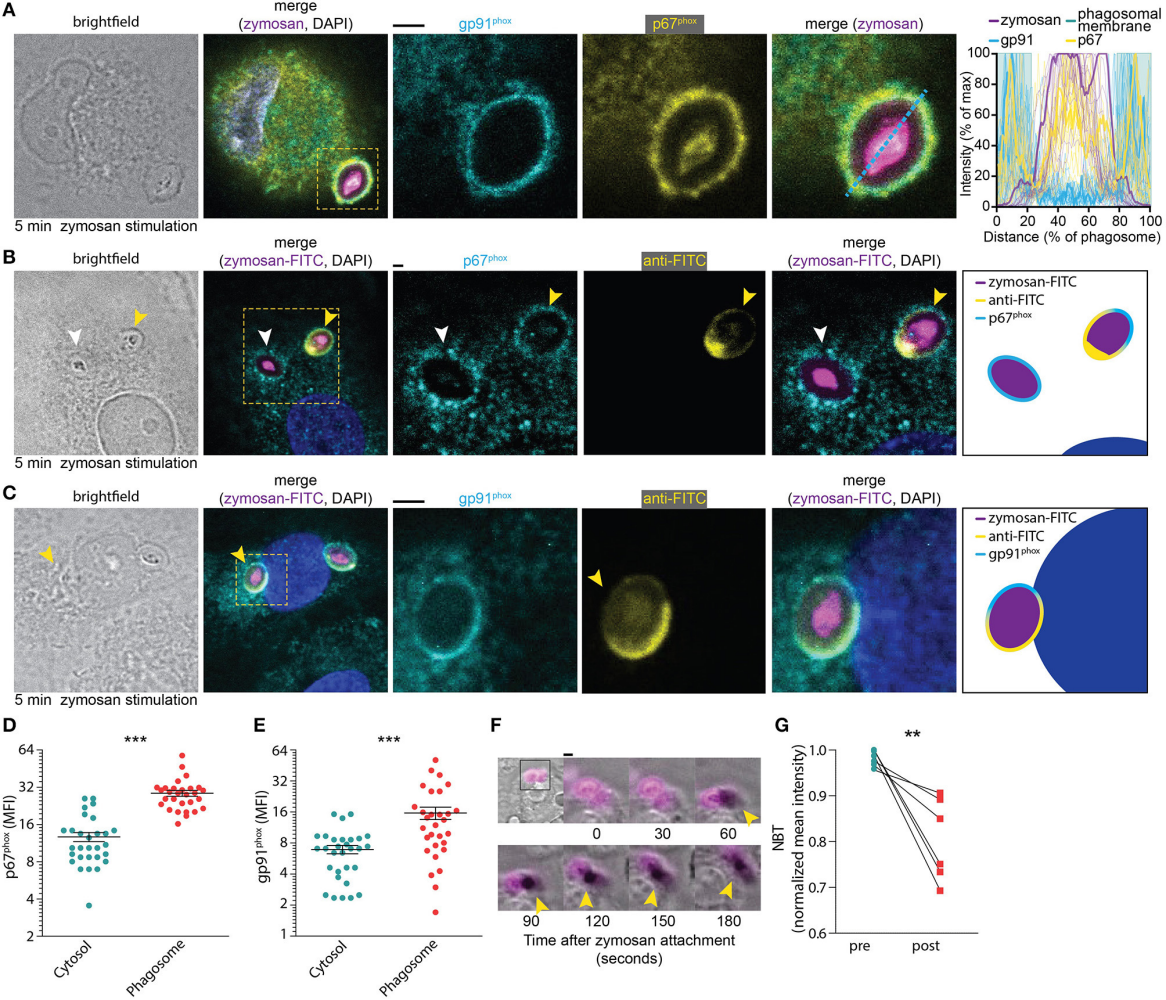
NOX2 assembly at the phagocytic cup in monocyte-derived dendritic cells. **(A)** Representative confocal micrograph of a cell pulsed for 5 min with Alexa Fluor 633-labeled zymosan (magenta in merge) and immunostained for gp91^phox^ (cyan), p67^phox^ (yellow), and DAPI (blue). Scale bar: 2 μm. Graph: fluorescence intensity cross-sections over zymosan particles for multiple phagosomes. The bold cross-sections are from the left-hand images (indicated with the dotted line). Twenty-one phagosomes from 3 donors were analyzed. **(B)** Representative confocal micrograph of cell pulsed with FITC-labeled zymosan (magenta in merge) and immunostained for anti-FITC (in absence of permeabilization; yellow), p67^phox^ (with permeabilization; cyan) and DAPI (blue). Yellow arrow head: phagocytic cup. White arrow head: closed phagosome. Note the localization of p67^phox^ at the phagocytic cup. Scale bar: 2 μm. Twenty-nine phagosomes from 15 cells and 3 donors were analyzed. **(C)** Same as **(B)**, but for gp91^phox^ (cyan). Scale bar: 2 μm. Thirty phagosomes from 16 cells and 3 donors were analyzed. **(D)** Mean fluorescent intensity (MFI) of p67^phox^ in the phagosome compared to the cytosol after 5 min of zymosan stimulation (as shown in **B**). Three donors were analyzed, each dot represents a single cell. *p* < 0.0001; paired Student's *t*-test. **(E)** Same as panel **(D)**, but now for gp91^phox^ as in panel **(C)**. *p* < 0.0001; paired Student's *t*-test. **(F)** Representative live cell epi-fluorescence imaging of a cell pulsed with zymosan labeled with both Alexa Fluor 633 (magenta) and nitrotetrazolium blue chloride (NBT) (transmission channel). Oxidation of NBT results in formation of dark formazan crystals (yellow arrow heads). Scale bar: 4 μm. Video file in Supplementary Movie 1. **(G)** Quantification of NBT crystal formation before and 25 min after attachment of a zymosan particle for six different donors (see also Supplementary Data 1). *p* = 0.009; paired Student's t-test. ^**^p < 0.01; ^***^p < 0.001.

We first determined the time needed for the assembly of NOX2 following phagocytosis. Both the membrane component gp91^phox^ and the cytosolic component p67^phox^, which is one of the last components to be added to the NOX2 complex for its assembly (29), were visualized by immunofluorescence labeling. A confocal microscopy time-series of zymosan incubation showed that these subunits already overlapped at the phagosomal membrane after 5 min (i.e., the earliest timepoint sampled after zymosan addition), confirming the rapid assembly of the NOX2 complex at phagosomes (Figure 1A). To assess whether NOX2 was assembled already at the nascent cup of emerging phagosomes, we also performed microscopy experiments with fluorescein isothiocyanate (FITC)-conjugated zymosan particles. Following incubation, we fixed the cells and stained with an anti-FITC antibody before permeabilization, as described earlier (30). This allowed us to visualize nascent cups, as the FITC staining is only present on zymosan particles that are not yet completely internalized. We observed assembly of NOX2 (i.e., the presence of gp91^phox^ and p67^phox^) already before closure of the phagocytic cup (Figures 1B–E), indicating that NOX2 is activated rapidly upon encountering a pathogen. In line with this, we also observed the presence of gp91^phox^ on ~75% of phagosomes positive for the early endosomal marker EEA1 (Supplemental Figure 1). These findings corroborate our previous findings that gp91^phox^ is recruited to nascent phagosomes from the plasma membrane (21, 28).

To obtain insight in the time scale of ROS production following zymosan uptake, we stimulated dendritic cells with fluorescently labeled zymosan together with nitrotetrazolium blue chloride (NBT). NBT forms insoluble formazan crystals upon oxidation and this can be visualized as dark deposits in the transmission channel during live cell imaging. We observed crystal formation within 2 min after attachment of a zymosan particle to a dendritic cell (Figure 1F; Supplementary Movie 1). This process continued for at least 25 min, after which most NBT was converted to formazan (Figure 1G). Together, these results show that NOX2 is assembled already at the nascent cup of emerging phagosomes and ROS production occurs rapidly (i.e., within minutes) after zymosan attachment.

**Figure 2:**
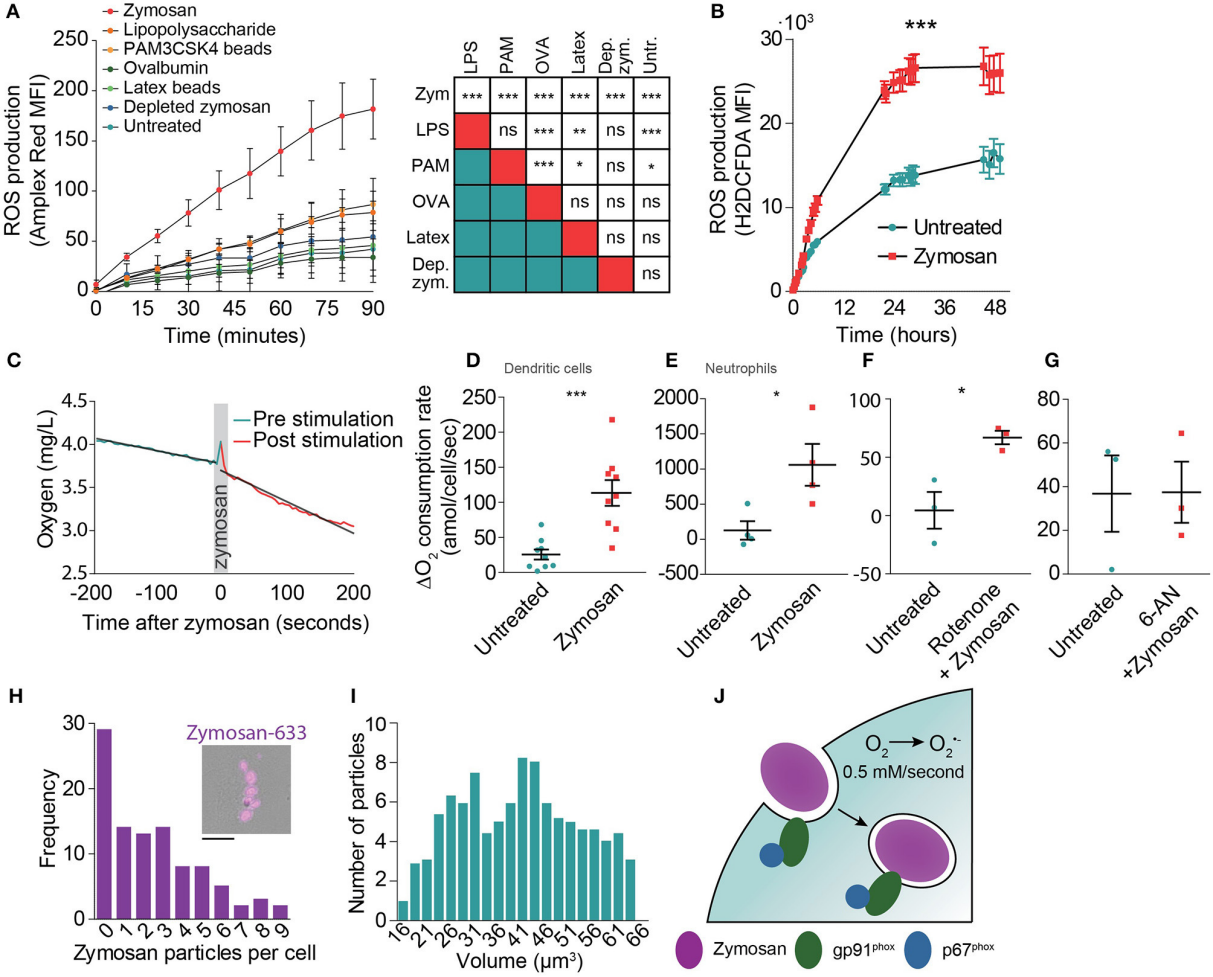
Determination of the oxygen consumption rate (OCR) of monocyte-derived dendritic cells following zymosan exposure. **(A)** ROS production by monocyte-derived dendritic cells exposed to a range of phagocytic cargoes and soluble TLR stimuli as determined by Amplex Red fluorescence. MFI: mean fluorescence intensity. Shown is the average of three donors ± SEM. The table shows results from linear regression analysis on the integrated curves (^***^p < 0.001; ^**^p < 0.005; ^*^p < 0.01). **(B)** ROS production by zymosan-exposed monocyte-derived dendritic cells as determined by H_2_DCFDA fluorescence. Shown is the average of three donors ± SEM. *p* < 0.0001; linear regression analysis on the integrated curves. **(C)** Temporal decline in oxygen levels in the culture medium before and after stimulation with zymosan in a closed container of a representative donor. **(D)** Difference in OCR of monocyte-derived dendritic cells before and after stimulation with zymosan in a closed container. Each data point presents an individual donor (*n* = 9). *p* = 0.0005; paired Student's *t*-test. **(E)** Same as panel **(D)**, but with neutrophils (*n* = 3). *p* = 0.0143; paired Student's *t*-test. **(F)** Difference in OCR in monocyte-derived dendritic cells before and after stimulation with zymosan with simultaneous addition of the mitochondrial complex I inhibitor Rotenone (*n* = 3). *p* = 0.0256; paired Student's *t*-test. **(G)** Difference in OCR before and after stimulation with zymosan in a closed container in cells pre-incubated for 1 h with the glucose-6-phosphate dehydrogenase inhibitor 6-aminonicotinamide (6-AN) (*n* = 3). *p* = 0.9868; paired Student's *t*-test. **(H)** Frequency distribution of the number of zymosan particles taken up by monocyte-derived dendritic cells following 30 min exposure to zymosan (99 cells pooled for three different donors; bin size: 1 particle). Insert: Representative epi-fluorescence microscopy image of a cell with phagocytosed Alexa Fluor 633-labeled zymosan (magenta). Scale bar: 10 μm **(I)** Frequency distribution of the volume of zymosan particles from microscopy (521 zymosan particles in total; bin size: 5 μm^3^). **(J)** Model figure of NOX2 activity during phagocytosis by monocyte-derived dendritic cells. NOX2 is already assembled and active before closure of the phagocytic cup and remains active on the phagosomal membrane for on average at least 10 h. NOX2-mediated conversion of oxygen (O_2_) to superoxide anion (O${}_{2}^{∙-}$) results in a production rate of ROS of approximately 0.5 mM/s.

Zymosan is a potent inducer of NOX2 activation (20, 21, 28) as it activates signaling of a number of pattern recognition receptors, including Toll-like receptor 2 (TLR2) and the C-type lectin Dectin-1 (31). Indeed, Amplex Red measurements showed that zymosan evoked the highest ROS production by monocyte-derived dendritic cells when compared to the soluble TLR agonist lipopolysaccharide (LPS; TLR4), ovalbumin (binds to the C-type lectin CD206) and to the other phagocytic cargoes PAM3CSK4-coated beads (TLR2) and depleted zymosan (only Dectin-1) (Figure 2A). In these experiments, the cells were all stimulated with the same amount of five particles per cell for the phagocytic cargoes: zymosan, PAM3CSK4-coated latex beads, depleted zymosan, and naked latex beads (negative control). For the soluble ligands LPS and ovalbumin, we could not directly relate their concentrations to that of large phagocytic particles and used final concentrations of 1 μg/ml for LPS and 2.5 μg/ml for ovalbumin. We also compared ROS production of human monocyte-derived dendritic cells with the murine cell lines JAWS-II and RAW264.7 using the intracellular fluorescent ROS probe H_2_DCFDA (32). JAWS-II is an immortalized cell line from mouse bone marrow and is a frequently used model system for dendritic cell function capable of phagocytosis and antigen presentation. RAW264.7 is derived from a murine tumor and is a frequently used model system for macrophages. Compared to monocyte-derived dendritic cells, zymosan resulted in ~40% less ROS production in RAW264.7 cells, whereas zymosan hardly induced any ROS production in JAWS-II cells (Supplemental Figure 2).

Next, we investigated the duration of ROS production to obtain an estimate of the total amount of ROS produced by human monocyte-derived dendritic cells. The duration of ROS production cannot be determined with OCR measurements, because in a closed chamber the amount of oxygen is limited, resulting in complete depletion of oxygen, whereas in an open chamber the oxygen levels are also influenced by an ingress of oxygen diffusion from the atmosphere which is difficult to quantify. We therefore measured ROS production of the monocyte-derived dendritic cells with H_2_DCFDA (32). Although this probe does not allow quantitative measurements of ROS levels, it does allow qualitative monitoring of ROS production for extended periods of time after stimulation with zymosan. ROS production was still observed at least 10 h post zymosan stimulation (Figure 2B). At later time points, the fluorescence increase of H_2_DCFDA in the zymosan exposed cells was similar to the non-stimulated control, indicating that zymosan-evoked ROS production had stopped. Flow cytometry measurements using the fixable viability marker Zombie Violet showed that about ~10% of the dendritic cells died during the first 10 h of zymosan incubation, whereas cell viability was not affected in absence of zymosan (Supplemental Figure 3).

Finally, to determine the OCRs during zymosan uptake, we measured real-time oxygen concentrations in the medium using a fiber optic sensor connected to a Fibox 3 LCD trace fiber-optic oxygen meter calibrated to 37°C. These measurements were done in a closed 1 ml chamber containing 1·10^6^ monocyte-derived dendritic cells. After reaching a stable baseline OCR, we added zymosan at a ratio of five particles/cell, triggering phagocytic uptake and ROS production. This caused an average OCR increase from 28 to 88 amol (1·10^−18^)/cell/second (Figures 2C,D), although there was a large variation among donors. This large variation in ROS production within the human population, which is likely due to genetic and life style differences, has been reported before (11) and is well-known for other immunological readouts such as cytokine secretion. Compared to monocyte-derived dendritic cells, a more than 10-fold higher rate of oxygen consumption was found in blood-isolated neutrophils following zymosan addition using the same methodology (Figure 2E; 1,059 amol/cell/s), which is in line with previous reports (5–7). To account for potential upregulation of cell metabolism following zymosan stimulation, we also treated monocyte-derived dendritic cells with the respiratory chain complex I inhibitor rotenone, which blocks oxidative phosphorylation (33). We found that the increase in OCR following zymosan addition was comparable to cells not treated with rotenone (Figure 2F; from 4 to 67 amol/cell/s). Conversely, inhibition of the pentose phosphate pathway using 6-aminonicotinamide (6-AN), which blocks generation of the NOX2 substrate NAD(P)H, completely abrogated zymosan-induced oxygen consumption (Figure 2G). These findings confirm the well-known role of NOX2 in TLR-evoked ROS production in human monocyte-derived dendritic cells (10, 11, 20, 21).

These results indicate that on average 60 amol superoxide/cell/s was produced following zymosan addition. As quantitative microscopy showed that ~3 zymosan particles were ingested per cell (Figure 2H), this means that ~20 amol/s of superoxide anion is produced per phagosome. Because most zymosan particles are ~40 μm^3^ in size (or ~40 fL) (Figure 2I), the average net superoxide anion production rate is approximately 0.5 mM/s within the phagosome. Combined with our estimation of the duration of ROS production by H_2_DCFDA measurements (Figure 2B), we estimate that the total amount of superoxide anion produced is ~720 fmol/phagosome, or about 4.2·10^11^ molecules/phagosome. Again, due to the large variation in phagocytic uptake among cells and oxygen consumption among donors (11), this number is only a coarse estimate and is expected to vary widely among cells and donors.

## Discussion

In this study, we estimated the production of ROS by NOX2 in zymosan-containing phagosomes of human monocyte-derived dendritic cells. By subtracting baseline oxygen consumption from total cellular oxygen consumption following zymosan addition, we estimate a ROS production of ~0.5 mM/phagosome/s. This estimate is based on the assumption that the majority of ROS is produced within phagosomes as supported by our finding that assembled NOX2 is mostly located at phagosomes. Moreover, we found that monocyte-derived dendritic cells assemble the NOX2 complex already at the nascent cup of phagosomes, allowing them to generate ROS within minutes upon encountering a pathogen, similar to neutrophils and macrophages (6) (Figure 2J). The presence of NOX2 already at the phagocytic cup is in line with observations from our (28, 34) and other (11) laboratories that NOX2 is assembled during phagosome formation, remains present during the early phase of phagosomal maturation (early markers EEA1 and phosphatidylinositol 3-phosphate) and is removed from phagosomes upon maturation to the late LAMP1 and V-ATPase positive stage. Unfortunately, direct quantitative measurements of ROS within the lumen of phagosomes are not technically feasible at present, but since our microscopy showed that assembled NOX2 was mainly present at phagosomal membranes, we consider our assumption that the majority of ROS is produced within phagosomes justified. Our estimated ROS production of ~0.5 mM/phagosome/s should be regarded as a coarse estimate, since there is large variation in terms of phagocytic activity and oxygen consumption among individual cells as well as between different donors [see also (11)]. In addition, our ROS measurements show that these numbers differ depending on the TLRs that are stimulated (Figure 2A). Nevertheless, despite being a coarse estimate, our results provide at least an order of magnitude of the ROS production in monocyte-derived dendritic cells.

Our data show that the ROS production in phagosomes of monocyte-derived dendritic cells is about an order of magnitude lower than the 5 mM/phagosome/s estimated for neutrophils (5–7). However, we found that ROS production in dendritic cells is sustained for ~10 h after uptake, which is at least an order of magnitude longer than in neutrophils and macrophages (5–7, 9). This means that although dendritic cells produce ROS at a lower rate, the cumulative ROS production over time in dendritic cells and neutrophils could be approximately similar. The viability of dendritic cells in this time frame is not much affected as ~90% of zymosan-pulsed cells survive. As noted in the introduction, both the lower, sustained ROS production and the maintained viability likely relate to the unique immune function of dendritic cells by promoting ROS-mediated MHC-I antigen cross-presentation to cytolytic T cells (11, 12, 17–23) and modulating the epitope repertoire for MHC-II presentation to helper T cells (24). The reduced activation of lysosomal proteases by ROS (12, 17, 18, 22, 23) could also allow the antigen to be preserved for the time that the dendritic cell needs to travel from a site of infection to the nearest lymph node to present antigen to T cells (35). The comparatively low but prolonged activity of NOX2 and their prolonged viability therefore directly contribute to the antigen presenting capacity of dendritic cells.

Given the role of phagosomal ROS in antigen presentation (11, 12, 17–24), it would be interesting to measure ROS production by the three major types of dendritic cells found in human peripheral blood: plasmacytoid dendritic cells and CD1c and CD141 positive myeloid dendritic cells (36). Plasmacytoid dendritic cells produce large amounts of type I interferons in response to microbial or viral infections and are key effectors of innate immunity and helper and cytolytic T cell priming (37). Both CD1c and CD141 positive myeloid dendritic cells can produce large amount of interleukin-12 to promote Th1 responses and cytolytic T cell priming. Both these myeloid dendritic cell subsets can also cross-present exogenous antigens to prime naive cytolytic T cells (38–43). It will also be interesting to measure ROS production by monocyte-derived macrophages, which, although their ROS production is lower compared to dendritic cells (11), are also capable of cross-presentation (44).

Next to antigen presentation, a second effect of NOX2 activity is metabolic reprogramming caused by the rapid consumption of both NADPH and oxygen (45, 46). An oxidative cellular redox state influences metabolism, and via this way promotes for example monocyte differentiation into pro-inflammatory effector cells (47). Moreover, ROS and its derivatives, particularly hydrogen peroxide and lipid aldehydes, are able to traverse membranes and signal inflammation in neighboring cells (48, 49). Thereby, ROS can amplify the inflammatory immune response, leading to production of more ROS. Thus, ROS have important functions in both innate and adaptive immune responses and having quantitative information on ROS generation is crucial for understanding dendritic cell function (45).

## Methods

### Cells

Dendritic cells were derived from human peripheral blood mononuclear cells (PBMCs) by IL-4 and GM-CSF as described previously (35). Neutrophils were isolated from heparinized venous blood by Ficoll-Histopaque (Sigma Aldrich, St. Louis, USA) density gradient centrifugation followed by hypotonic erythrocyte lysis (50). Buffy coats and whole blood were obtained as anonymous coded specimens from the Dutch blood bank (Sanquin) and were handled according to known practice and legal guidelines. The research with human blood samples at the Department of Tumor Immunology complies with all institutional and national ethics regulations and has been approved by the ethics committee of Sanquin. All blood donors were informed of the research and have granted their consent. The age range of the donors was between 30 and 60 years. For OCR measurements, cells were cultured in phenol-red free RPMI-1640 (Thermo Fisher Scientific, Waltham, USA). JAWS-II (American Type Culture Collection (ATCC), CRl-11904) were cultured in MEM Alpha (Thermo Fisher Scientific) supplemented with 20% FBS (Greiner Bio-one, Kremsmünster, Austria), 5 mM UltraGlutamine (Lonza, Basel, Switzerland), 1 mM sodium pyruvate (Thermo Fisher Scientific), 0.05 mM 2-mercaptoethanol, 10 ng/ml recombinant mouse GM-CSF, and 1% antibiotic-antimycotic (Thermo Fisher Scientific). RAW264.7 (ATCC, TIV-71) cells were cultured in Dulbecco's Modified Eagle Medium (DMEM) (Thermo Fisher) supplemented with 10% FBS (Greiner Bio-one), 2 mM UltraGlutamine (Lonza), and 1% antibiotic-antimycotic (Thermo Fisher Scientific). Cells were stimulated with zymosan A (from *Saccharomyces cerevisiae*, Sigma-Aldrich, St. Louis, USA; five particles/cell), LPS (Lipopolysaccharides from Escherichia coli O111:B4; Sigma Aldrich; final concentration of 1 μg/ml), PAM3CSK4 (InvivoGen, Toulouse, France) coated latex beads (five beads/cell), depleted zymosan (InvivoGen; five particles/cell), naked latex beads (five beads/cell), soluble ovalbumin (final concentration of 2.5 μg/ml; Endofit ovalbumin, InvivoGen) or Alexa Fluor 633-labeled zymosan A in serum-free RPMI-1640 (five particles/cell). To inhibit NOX2 activity by blocking generation of NAD(P)H, cells were pre-incubated for 1 h with 10 μM 6-aminonicotinamide (Sigma Aldrich). To inhibit mitochondrial respiration by inhibition of respiratory complex I, rotenone (Sigma Aldrich) was added simultaneously with the zymosan stimulus to a final concentration of 500 nM.

### H_2_DCFDA Measurements

Cells were loaded for 10 min with 10 μg/ml 2′,7′-dichlorodihydrofluorescein diacetate (H_2_DCFDA, Thermo Fisher Scientific) in DMSO, stimulated with five particles/cell of zymosan and fluorescence (ex: 485/20, em: 530/30) was measured using a CytoFluor II microplate reader (Thermo Fischer Scientific). Background fluorescence was subtracted from all measurements.

### Amplex Red Measurements

Fifty thousand monocyte-derived dendritic cells were stimulated with the stimuli specified above and plated in individual wells of a clear flat-bottom 96-wells plate. Cells were subsequently incubated for 1 h at room temperature. After incubation, supernatant was removed and cells were washed once with phosphate-buffered saline (PBS). Fifty microliter Amplex Red (Thermo Fisher Scientific) mix (25 μM Amplex Red and 0.1 U HRP in phosphate-buffered saline) was added to each well. Amplex Red fluorescence was measured every 10 min using a CytoFluor II microplate reader (excitation: 530/25, emission: 590/35) (Thermo Fisher Scientific).

### Microscopy

Live cell imaging was performed on a Leica DMI-6000B inverted epi-fluorescence microscope using a Leica HCX PL APO 40x/0.85 objective. NOX2 assembly was imaged on a Leica SP8 confocal using a Leica HC PL APO CS2 63x/1.2 water immersion objective. For immunofluorescence, cells were fixed with 4% paraformaldehyde in PBS for 20 min, then permeabilized with 0.1% saponin in PBS. NOX2 subunits were stained using the following antibodies: mouse monoclonal IgG1 anti-CYBB (1:200; D162-3 MBL International, Woburn, USA), rabbit polyclonal IgG anti-gp91 (1:100; ab180642 Abcam, Cambridge, UK) and rabbit polyclonal IgG anti-p67 (1:500; 07-002 Millipore-Sigma, Burlington, MA, USA). EEA1 was stained with mouse monoclonal IgG1 anti-EEA1 (1:100; 610456 BD Biosciences, San Jose, CA, USA). Coverslips were mounted in mounting medium containing 0.01% Trolox (6-hydroxy-2,5,7,8-tetramethylchroman-2-carboxylic acid) and 68% glycerol in 200 mM sodium phosphate buffer at pH 7.5 with 0.1 μg/ml DAPI. Recruitment of NOX2 subunits gp91^phox^ and p67^phox^ was quantified by comparing fluorescent intensities in the cytosolic regions to phagosomal membrane regions selected by thresholding for fluorescent zymosan particles partially or completely negative for FITC.

### NBT Assay

Cells were incubated with 12 μg/ml NBT (Hoffmann-La Roche, Rish-Rotkreuz, Switzerland) in Live Cell Imaging Solution (Thermo Fisher Scientific) and stimulated with Alexa Fluor 633-labeled Zymosan A (five particles/cell). Formazan crystal formation was quantified in regions of interest by thresholding on the fluorescence of the zymosan particles using a custom imageJ script (Supplementary Data 1) and measuring the intensities of these regions of interest in the bright-field channel. As NBT crystals are dark, pixel intensity decreases upon formation of NBT crystals and we normalized all values to those measured in the first frame.

### OCR Measurements

The OCR during zymosan uptake was measured with 1·10^6^ cells in a closed 1 ml chamber, using a fiber optic trace oxygen meter (PreSens Precision Sensing, Regensburg, Germany) with an Oxygen Sensor Spot (PreSens) placed inside. Measurement conditions were 37°C and 1 atm. Upon reaching a steady baseline OCR, 5 zymosan particles/cell were added by injection.

### Viability Assay

Cells were washed with PBS and incubated for 30 min with Zombie Violet fixable viability dye (1:2000 in PBS) (BioLegend, San Diego, CA, USA) in a V-bottom 96-well plate at 100,000 cells per well. Cells were then washed and fixed for 5 min in 4% paraformaldehyde in PBS and analyzed on a FACS Lyric flow cytometer (BD Biosciences).

### Statistical Analysis

Paired Student's *t*-tests were applied to assess significance, unless indicated otherwise. For multiple comparisons, a repeated measures ANOVA with Bonferroni *post-hoc* testing was applied. For the H_2_DCFDA time course experiments and the comparison between various TLR stimuli, we integrated the curves and compared the slopes using linear regression analysis in Graphpad Prism 5.

## Data Availability

The datasets generated for this study are available on request to the corresponding author.

## Author Contributions

LP, ID, PL, and GB designed and performed the experiments. AS contributed to the neutrophil experiments. SC and WV contributed to the oxygen consumption experiments. All authors contributed to writing the manuscript.

### Conflict of Interest Statement

The authors declare that the research was conducted in the absence of any commercial or financial relationships that could be construed as a potential conflict of interest.

## Supplementary Material

Supplementary Movie 1 Time-lapse microscopy of a monocyte-derived dendritic cell (bright-field) taking up a fluorescently labeled zymosan particle (magenta). Deposition of formazan crystals by oxidation of NBT can be observed as dark spots in the bright-field channel. Timestamp in minutes:seconds.

Supplementary Data 1 ImageJ script used for analysis of formazan crystal formation.

Supplemental Figure 1 gp91^phox^ is present on EEA1-positive phagosomes. Representative confocal micrograph of monocyte-derived dendritic cell pulsed with Alexa fluor 633-labeled zymosan (magenta in merge) for 5 min and immunostained for gp91^phox^ (cyan) and EEA1 (yellow). Arrowhead indicates a phagosome. The graph shows EEA1-positive phagosomes that were blindly assessed manually as positive or negative for gp91^phox^ (*n* = 3, at least 40 phagosomes per donor). *p* = 0.0207; paired Student's *t*-test. ^*^p < 0.05.

Supplemental Figure 2 ROS production by mouse phagocytic cell lines. Time course experiments following stimulation with 5 zymosan particles per cell. ROS production was measured by H_2_DCFDA fluorescence in human monocyte-derived dendritic cells, mouse RAW264.7 macrophages, and mouse JAWS-II dendritic cells. MFI, mean fluorescence intensity (*n* = 3 ± SEM).

Supplemental Figure 3 Viability of monocyte-derived dendritic cells stimulated with zymosan for 48 h. Representative dot plots showing forward scatter vs. Zombie Violet fluorescence intensity of monocyte-derived dendritic cells incubated with 5 zymosan particles per cell for the indicated durations. Smaller dot plots show back gating of lymphocyte population gate based on forward and side scatter. The scatter plot shows percentages of Zombie Violet positive cells for the indicated time points (*n* = 3). Repeated measures ANOVA with Bonferroni *post-hoc* testing (^**^*p* < 0.005; ^*^*p* < 0.05).
