# Supplementary material for: Human Monocyte-Derived Dendritic Cells Produce Millimolar Concentrations of ROS in Phagosomes Per Second
Source: Front Immunol. 2019 May 29;10:1216. doi: 10.3389/fimmu.2019.01216 (PMC6548834; doi:10.3389/fimmu.2019.01216)
Supplement: Supplementary Data 1 — ImageJ script used for analysis of formazan crystal formation. [file Data_Sheet_1.docx]

**Supplementary Data 1**

**ImageJ script used for analysis of formazan crystal formation**

windowname = getTitle();

roiManager("Reset");

//run("Clear Results");
 //Comment this command back in to clear the results window before each measurement.

/* Duplicates the channel containing zymosan 633 emission (channel 2).

* Runs a user-defined threshold to generate ROIs for zymosan-633 positive particles.

* Includes particles sized 12 square micrometer and up.

*/

run("Channels Tool...");

Stack.setDisplayMode("color");

run("Duplicate...", "duplicate channels=2");
 // Channel 2 contains zymosan-633 signal.

setAutoThreshold("Default dark");

run("Threshold...");

waitForUser("set appropriate threshold");

setOption("BlackBackground", false);

run("Convert to Mask", "method=Default background=Dark calculate");

run("Analyze Particles...", "size=12-infinity include add stack");

close();

/* Fits an ellipse around every ROI and enlarges it to include NBT signal in the entire phagosome.

* Then, measures MFI in transmission channel (channel 1) based on ROIs.

*/

run("Set Measurements...", "area mean standard display redirect=None decimal=3");

selectWindow(windowname);

Stack.setChannel(1); //Channel 1 contains bright field image.

count = roiManager("count");

for (j=0; j<count; j++){

roiManager("select", j);

run("Fit Ellipse");

run("Enlarge...", "enlarge=1"); //Enlarges ellipse by 1 micron.

run("Measure");

}
