# Supplementary figures and images for: Human Monocyte-Derived Dendritic Cells Produce Millimolar Concentrations of ROS in Phagosomes Per Second
Source: Front Immunol. 2019 May 29;10:1216. doi: 10.3389/fimmu.2019.01216 (PMC6548834; doi:10.3389/fimmu.2019.01216)

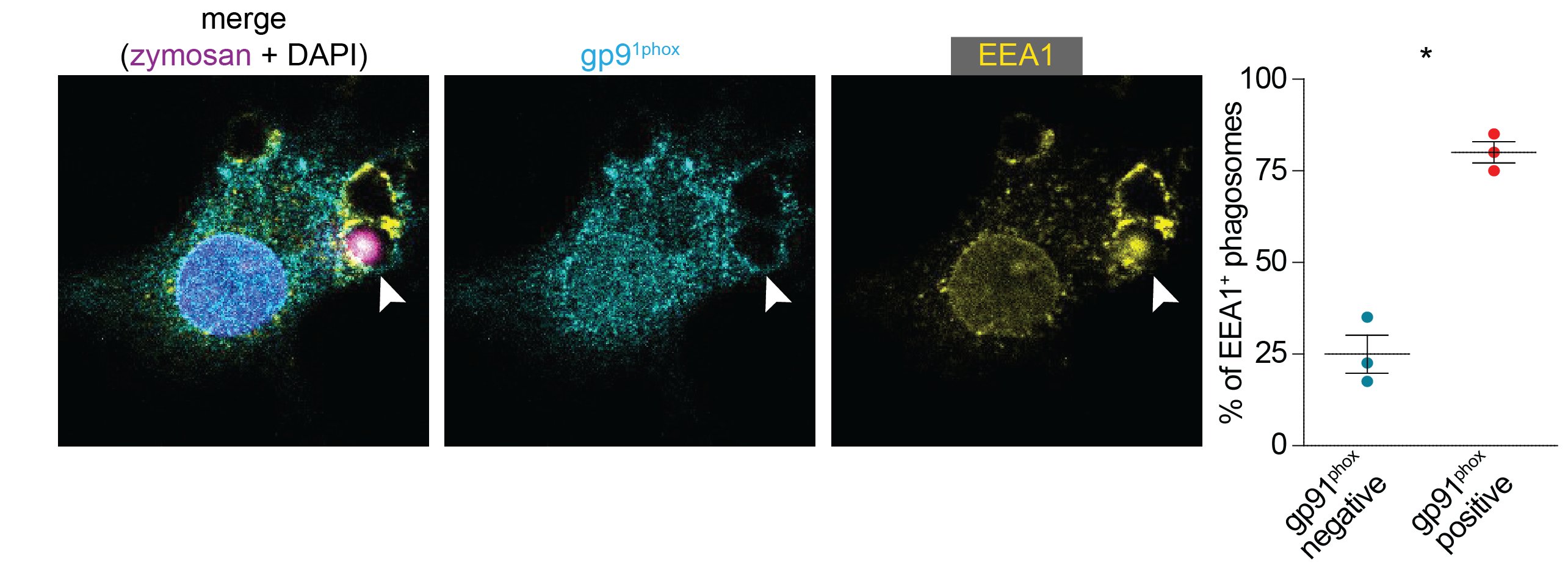

Supplement: Supplemental Figure 1 — gp91phox is present on EEA1-positive phagosomes. Representative confocal micrograph of monocyte-derived dendritic cell pulsed with Alexa fluor 633-labeled zymosan (magenta in merge) for 5 min and immunostained for gp91phox (cyan) and EEA1 (yellow). Arrowhead indicates a phagosome. The graph shows EEA1-positive phagosomes that were blindly assessed manually as positive or negative for gp91phox (n = 3, at least 40 phagosomes per donor). p = 0.0207; paired Student's t-test. *p < 0.05. [file Image_1.PNG]

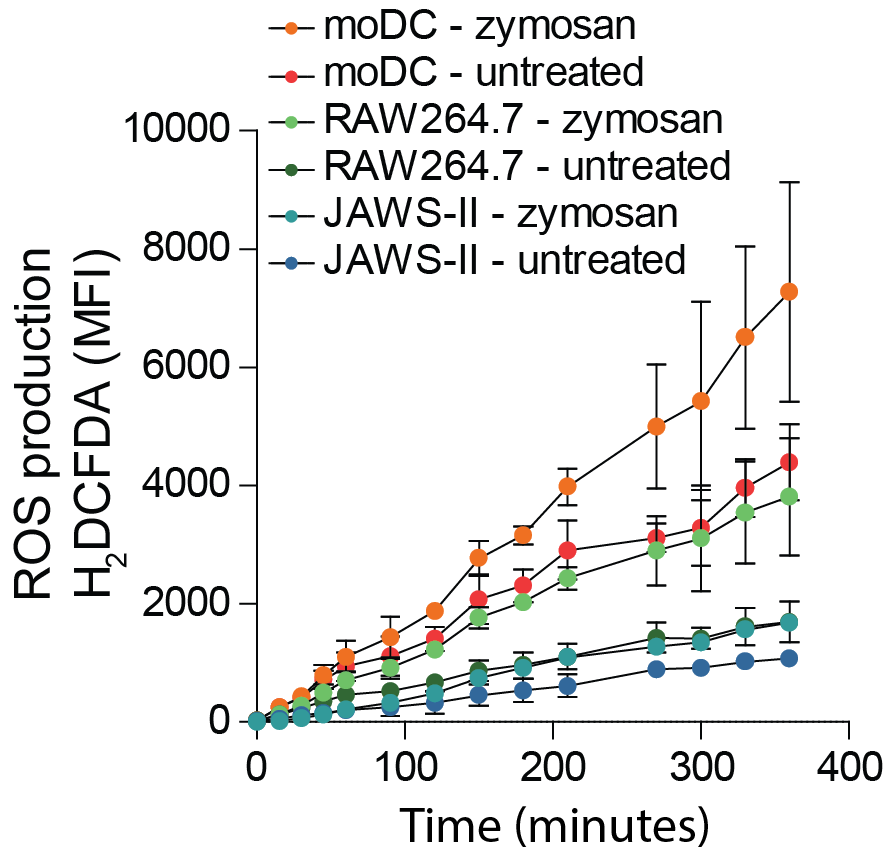

Supplement: Supplemental Figure 2 — ROS production by mouse phagocytic cell lines. Time course experiments following stimulation with 5 zymosan particles per cell. ROS production was measured by H2DCFDA fluorescence in human monocyte-derived dendritic cells, mouse RAW264.7 macrophages, and mouse JAWS-II dendritic cells. MFI, mean fluorescence intensity (n = 3 ± SEM). [file Image_2.PNG]

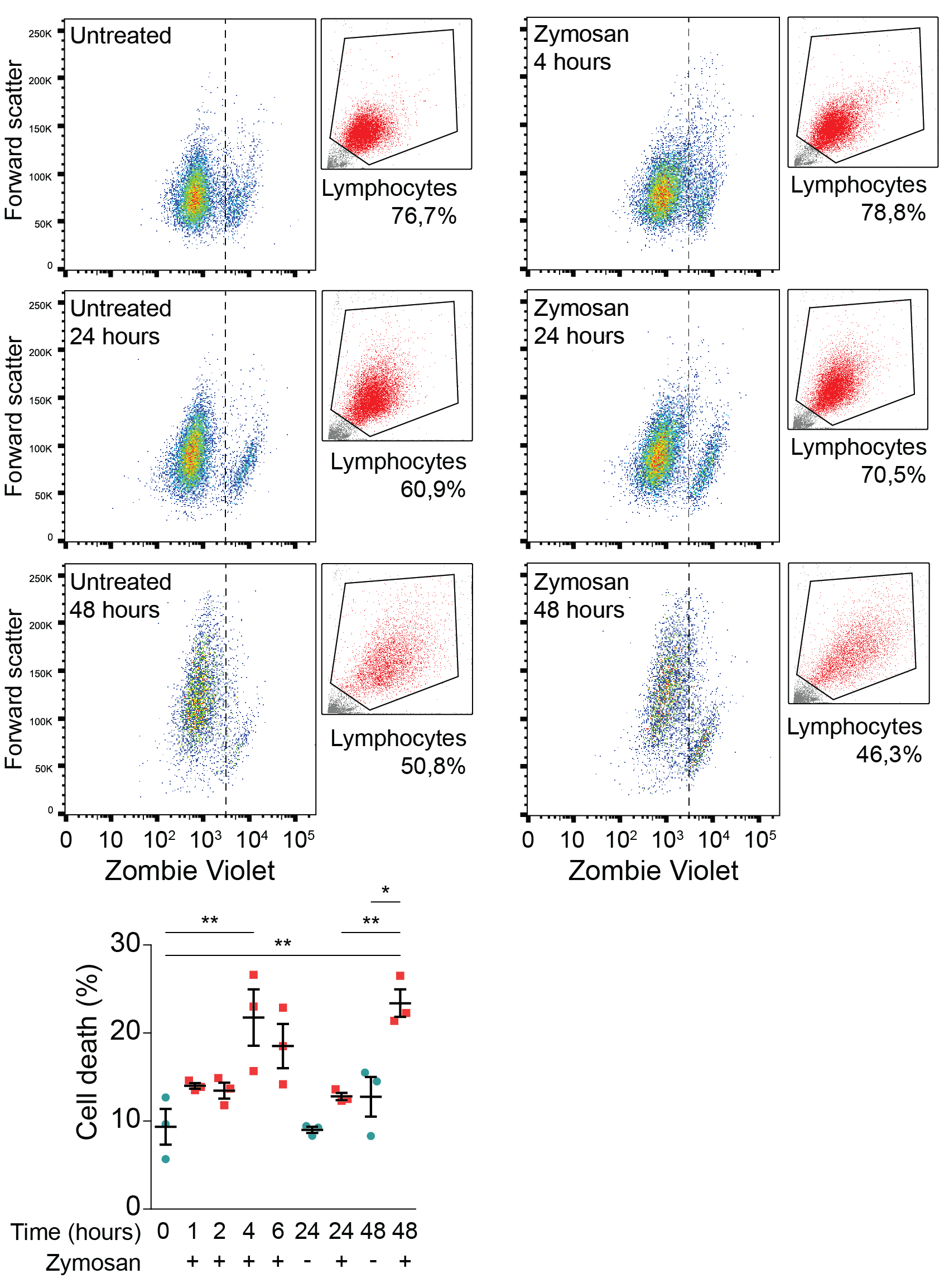

Supplement: Supplemental Figure 3 — Viability of monocyte-derived dendritic cells stimulated with zymosan for 48 h. Representative dot plots showing forward scatter vs. Zombie Violet fluorescence intensity of monocyte-derived dendritic cells incubated with 5 zymosan particles per cell for the indicated durations. Smaller dot plots show back gating of lymphocyte population gate based on forward and side scatter. The scatter plot shows percentages of Zombie Violet positive cells for the indicated time points (n = 3). Repeated measures ANOVA with Bonferroni post-hoc testing (**p < 0.005; *p < 0.05). [file Image_3.PNG]
